# Supplementary material for: Increased Prevalence of Headaches and Migraine in Patients with Psoriatic Arthritis and Axial Spondyloarthritis: Insights from an Italian Cohort Study
Source: Biomedicines. 2024 Feb 5;12(2):371. doi: 10.3390/biomedicines12020371 (PMC10886921; doi:10.3390/biomedicines12020371)
Supplement: Supplementary file 1 [file biomedicines-12-00371-s001.zip › biomedicines-2667617-supplementary.pdf]

Article

Increased prevalence of headaches and migraine in patients with Psoriatic Arthritis and Axial Spondyloarthritis: Insights from an Italian Cohort Study

Supplementary Material

Table S1. Comparison PsA and healthy controls

	**Entire PsA Population (216)**	**Healty controls (87)**	**p**
Age (years)	57 (49-63)	56 (43-68)	0.9
Gender	34.26% m<br>75.66% f	36.78% m<br>63.22% f	0.7
BMI	26.5 (23.7-29.9)	26.0 (24-27.8)	0.09
Charlson Comorbidity Index	2 (1-3)	2 (0-3)	0.8

Table S2. Comparison axSpA and healthy controls

	**axSpA Population 70 (100%)**	**Healty controls (87)**	**p**
Age (years)	57 (48-66)	56 (43-68)	0.9
Gender	42.86% m<br>57.14% f	36.78% m<br>63.22% f	0.4
BMI	25.76 (23.8- 28.05)	26.0 (24-27.8)	0.7
Charlson Comorbidity Index	2 (1-3)	2 (0-3)	0.3
